# Pressure-Driven Spin Crossover Involving Polyhedral Transformation in Layered Perovskite Cobalt Oxyfluoride

**DOI:** 10.1038/srep36253

**Published:** 2016-11-02

**Authors:** Yoshihiro Tsujimoto, Satoshi Nakano, Naoki Ishimatsu, Masaichiro Mizumaki, Naomi Kawamura, Takateru Kawakami, Yoshitaka Matsushita, Kazunari Yamaura

**Affiliations:** 1 Research Institute for Functional Materials, National Institute for Materials Science, 1-1 Namiki, Ibaraki 305-0044, Japan; 2 Graduate School of Science, Hiroshima University, 1-3-1 Kagamiyama, Higashi-Hiroshima 739-8526, Japan; 3 Japan Synchrotron Radiation Research Institute, 1-1-1 Kouto, Sayo-cho, Sayo-gun, Hyogo 679-5198, Japan; 4 Department of Physics, College of Humanities and Sciences, Nihon University, Sakurajosui, Setagaya-ku, Tokyo 156-8550, Japan; 5 Research Network and Facility Services Division, National Institute for Materials Science, 1-2-1 Sengen, Ibaraki 305-0047, Japan

## Abstract

We report a novel pressure-driven spin crossover in layered cobalt oxyfluoride Sr_2_CoO_3_F with a distorted CoO_5_ square pyramid loosely bound with a fluoride ion. Upon increasing pressure, the spin state of the Co(III) cation gradually changes from a high spin state (*S* = 2) to a low spin state (*S* = 0) accompanied by a anomalously large volume contraction (bulk modulus, 76.8(5) GPa). The spin state change occurs on the CoO_5_ pyramid in a wide pressure range, but the concomitant gradual shrinkage of the Co–F bond length with pressure gives rise to a polyhedral transformation to the CoO_5_F octahedron without a structural phase transition, leading to the full conversion to the LS state at 12 GPa. The present results provide new effective strategy to fine-tune electronic properties of mixed anion systems by controlling the covalency in metal-ligand bonds under pressure.

Transition metals with electronic configurations from *d*^4^ to *d*^7^ take either the high spin (HS) or low spin (LS) state as result of a competition between the Hund coupling (intra-atomic exchange energy) and the crystal field energy favoring higher and lower spin configurations, respectively. Spin crossover or spin state transition can be induced by controlling such a subtle balance with some external perturbation, for example, temperature, light-irradiation, and pressure, as exemplified in a wide range of materials from metal oxides through organometallic molecules to haemoglobin^1^,^2^,^3^,^4^,^5^. The bistability is of great interest for potential applications as memory devices.

While the variety of metal centers which exhibits a spin crossover is mostly limited to *d*^6^ Fe(II) with octahedral coordination^6^,^7^, little is known about iso-electronic Co(III) spin crossover systems regardless of types of coordination geometry, with the exception of LaCoO_3_ with perovskite structure^8^,^9^,^10^ and coordination complexes [CoL_2_PF_6_ (L = {(C_5_H_5_)Co[PO(OC_2_H_5_)_2_]_3_}^−^)^11^ which show thermally induced spin crossover from LS to HS state. The pressure-induced spin state transition is also observed in the former^12^,^13^. Given the fact that octahedrally coordinated Co(III) cation exclusively takes the LS state because of the relatively weak intra-atomic energy and strong crystal field^14^, modification of octahedral symmetry around the metal center which reduces the crystal field splitting is essential so as to make two spin multiplicity close in energy. However, in comparison with coordination complexes adopting rich variety of ligands, the selectivity of ligands is highly restricted in metal oxides. In fact, the approach frequently employed is cation substitution that indirectly distorts octahedral symmetry, as in 6-coordinated Pr_0.5_Ca_0.5_CoO_3_^15^,^16^ and 5-coordinated BiCoO_3_^17^,^18^. In this context, the study on the effect of anion substitution on spin state changes is of value for further understanding the chemistry of Co(III) cation.

Recent development of synthetic techniques for non-molecular solids enables us to design unprecedented coordination environment that cannot be obtained by a conventional solid state reaction. One such examples is mixed anion system, which offers good opportunities for new physical or chemical properties caused by different covalency, ionic sizes, oxidation states between oxygen and other anions. For example, oxynitride MnTaO_2_N showing a helical spin order^19^ and oxyhydride SrCrO_2_H with a high Néel temperature^20^ was synthesized using a high-pressure method, while the superconducting cuprate Sr_2_CuO_2_F_2+δ_^21^ and hydrid-ion conductors LaSrCoO_3_H_0.7_^22^ and BaTiO_3-*x*_H_*x*_ (*x* ~ 0.6)^23^ were obtained by low-temperature topotactic reactions. Despite a growing number of mixed anion compounds over the last two decades, however, the anion-lattice engineering has never been applied to studying on spin crossover phenomena. Here we report a novel pressure-induced spin state change involving a polyhedral transformation in the layered cobalt oxyfluoride antiferromagnet Sr_2_CoO_3_F with the space group *I*4/*mmm* (Fig. 1), which was previously synthesized by high pressure reaction^24^. The oxyfluoride adopts so-called K_2_NiF_4_-type structure, but a preferential occupation of the apical anion sites by F anion equally with O anion in a random manner leads to the Co-site off-centering to 4*e*(0, 0, *z*). The strong covalency of oxide ion in comparison with fluoride ion yields a distorted square pyramid of CoO_5_ loosely bound by one F anion at ambient pressure, and then the Co^3+^ cation takes the HS state with the electronic configuration of (*d*_*xy*_)^2^(*d*_*xz*_, *d*_*yz*_)^2^(*d*_*x*_^2^−_*y*_^2^)^1^(*d*_*z*_^2^)^1^ 25. Our high pressure study has demonstrated that a continuous depopulation of the HS state into a LS state occurs on the CoO_5_ pyramid in an extended pressure range, but a gradual shrinkage of the Co–F bond length with pressure results in the formation of the CoO_5_F octahedron, leading to the full conversion to the LS state at 12 GPa.

## Results and Discussion

Sr_2_CoO_3_F was prepared according to ref. 24. The cell parameters of the product at ambient pressure were *a*_0_ = 3.8309(11) Å, *c*_0_ = 13.217(4) Å, and *V*_0_ = 193.9(1) Å^3^, which are consistent with the previous report^24^,^25^. Figure 2a shows the pressure evolution of the SXRD patterns between 0.7 and 15.3 GPa for Sr_2_CoO_3_F. The SXRD data revealed that Sr_2_CoO_3_F adopts the space group *I*4/*mmm* in the whole pressure region. No anomaly associated with structural symmetry change from *I*4/*mmm* was observed within the experimental resolution. However, the observed diffraction peaks gradually shifted to higher 2*θ* angle with increasing pressure, indicative of a volume contraction. Figure 2b,c show the pressure dependences of the normalized lattice constants (*a*/*a*_0_ and *c*/*c*_0_) and the volume, where *a*_0_ and *c*_0_ are the lattice constants at ambient pressure. No pressure hysteresis was observed in the cell parameters at intervals of one giga-pascal. The volume apparently exhibited a monotonic decrease with increasing pressure up to 15.3 GPa. We analyzed the data by the Birch-Murnaghan equation of state^26^ expressed as


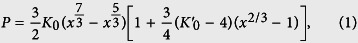


where *x*, *K*_0_, and *K′*_0_ represent *V*_0_/*V*, the bulk modulus, and its pressure derivative at ambient pressure, respectively. *K′*_0_ was fixed to 4, which is a value widely adopted in solid state materials. The fitting gave *K*_0_ = 76.8(5) GPa, much smaller than those observed in isostructural layered oxides with the metal center being octahedrally coordinated, such as Sr_2_MnO_4_ (*K*_0_ = 129 GPa)^27^, La_2_CuO_4_ (*K*_0_ = 181 GPa)^28^. As far as we know, Sr_2_CoO_3_F is the most compressible in related layered perovskite compounds. The unusually large volume contraction is reminiscent of LaCoO_3_ perovskite with *K*_0_ = 150(2) GPa^12^. Its bulk modulus is also much smaller than those of related perovskite oxides with the same octahedral tilt system (for example, *K*_0_ = 190 GPa for LaAlO_3_^29^).

In comparison with the volume change, high pressure effects on the structure of the oxyfluoride are pronounced in the *a* and *c* axes. Both the normalized lattice parameters decreased isotropically with decreasing pressure up to 6.4 GPa. The linear compressibility expressed by [*β*_*L*_ = (−1/*L*)(*δL*/*δP*)_*T*_, is estimated to be *β*_*a*_^*LP*^ = 3.54(5) × 10^−3 ^GPa^−1^ and *β*_*c*_^*LP*^  = 3.71(3) × 10^−3^ GPa^−1^ in the range of 0–6.4 GPa (low *P* region). As expected from the high *K*_0_ value, both of the compressibilities are significantly larger than *β*_*a*_ ≈ *β*_*c*_  = 2.2 × 10^−3^ GPa^−1^ for Sr_2_MnO_4_^27^ and *β*_*a*_ = 1.37(5) × 10^−3^ GPa^−1^, *β*_*b*_ = 2.0 × 10^−3^ GPa^−1^, and *β*_*c*_ = 1.6 × 10^−3^ GPa^−1^ for La_2_CuO_4_^28^. Further increase in pressure above 7 GPa led to anisotropic lattice expansion. The values of *β*_*a*_^*HP*^ and *β*_*c*_^*HP*^ in the *P* range from 11.3 to 15.3 GPa (high *P* region) are 1.37(5) × 10^−3^ GPa^−1^ and 4.27(2) × 10^−3^ GPa^−1^, respectively. Here, it is noteworthy that the value of *β*_*a*_^*HP*^ is less than half of that of *β*_*a*_^*LP*^ whereas the high compressibility along the *c* axis is maintained in the whole pressure region. These behaviors cannot be explained simply by a reduction in the spin state or ionic radius of the Co ion. As discussed later, the difference between the bonding characters of apical oxide and fluoride ions results in the anisotropic volume contraction.

*Kβ* XES involving 3*p*→1*s* transitions is a very useful probe to detect the local unpaired electrons in the 3*d* orbitals, namely the localized magnetic moments. Although XES contains numerous terms such as intra-atomic multiplet effects, charge-transfer effects and spin-orbit interaction^13^,^30^,^31^, variation from a HS to a LS state is characterized by a suppression of the relative intensity of a low-energy satellite *Kβ’* to the main emission *Kβ*_1,3_, and a lower-energy shift of the main peak position. Figure 3a shows the pressure evolution of the Co *Kβ* emission spectra up to 12 GPa at room temperature. All the spectra are normalized to the spectral area. In Fig. 3b, difference spectra with respect to the 1 GPa spectrum are displayed. The spectrum at 1 GPa clearly shows the main emission *Kβ*_1,3_ and its low-energy satellite *Kβ’* peaks centered at around 7.650 and 7.637 keV, respectively, which are consistent with the HS state confirmed by the neutron diffraction studies^15^. Application of pressure resulted in a gradual decrease (increase) in the intensities of the *Kβ’* (*Kβ*_1,3_) peaks and a spectral shift of the main emission to lower energy. These behaviors correspond well to what is expected from spin state changes. To qualitatively evaluate the spin state change, the integrals of the absolute values of the difference spectra (IAD) were calculated. The IAD_*i*_ value for the spectrum at a given pressure (*I*_*i*_) with respect to a reference spectrum (*I*_*ref*_) is expressed as the following equation


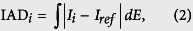


where *I*_*i*_ and *I*_*ref*_ are intensities normalized by the area. The IAD value is known to change linearly with the magnitude of the localized spin moments: the IAD value for |*ΔS*| = 2 is 0.12^13^. Figure 3c shows the pressure evolution of the IAD integrated in 7.61−7.67 keV for Sr_2_CoO_3_F. The XES data at 1 GPa was used as the reference spectrum for the full HS state. The IAD values increased gradually with pressure and reached 0.12 at 12 GPa (= *P*_s_), indicative of a continuous and complete depopulation of the HS state to a LS state. There were no anomaly associated with an IS state at pressures corresponding to |*ΔS*| = 1. The XES spectrum at 1 GPa under decompression is shown in Supplementary Figure S1. The intensity of the satellite peak and the main peak position were entirely recovered in the decompression process, which indicates that the HS-to-LS state change is reversible. The non-magnetic *S* = 0 sate is also confirmed in the electrical resistance measurements under pressure. As shown in Fig. 4, the electrical resistance (*R*) at room temperature gradually decreased with increasing pressure but upturned at around 8 GPa. The *R* value at 24 GPa is on the order of 10^6^ ohm, which strongly suggests that the semiconducting state persists in the measured pressure range. This upturn behavior can be attributed to the dominant population of the LS state as seen from the IAD vs *P* plot in Fig. 3c. The inset of Fig. 4 presents the temperature dependence of electrical resistance normalized by *R*_310 K_. Both the *R*(*T*) curves at 3.4 and 10.8 GPa increased with decreasing temperature, and the *R* values below 280 K were too high to be measured. No sign of metallization was observed in the measured temperature and pressure ranges.

**Figure 1:**
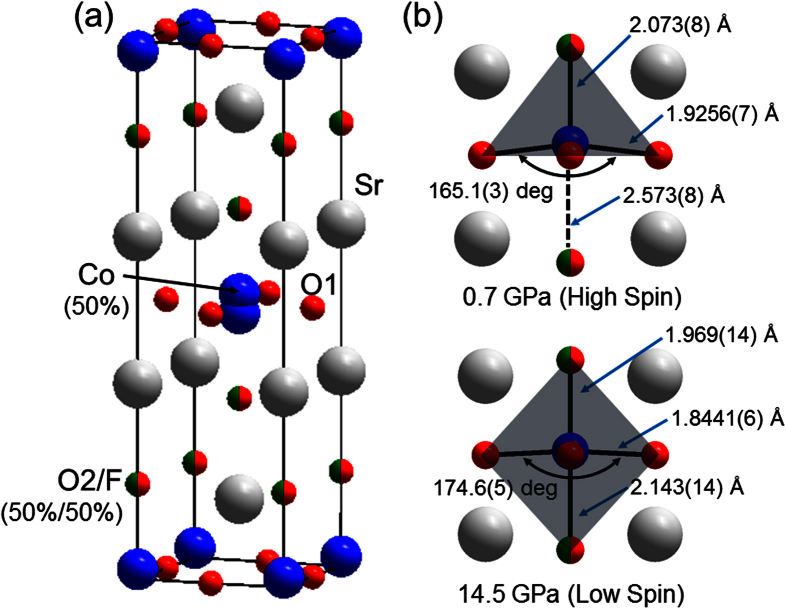
(**a**) The crystal structure of Sr_2_CoO_3_F at ambient pressure. Blue, red, green, and grey spheres represent Co, O, F, and Sr atoms, respectively. (**b**) Local coordination environment around Co center at 0.7 and 14.5 GPa. The gray shade represents the coordination polyhedron.

**Figure 2:**
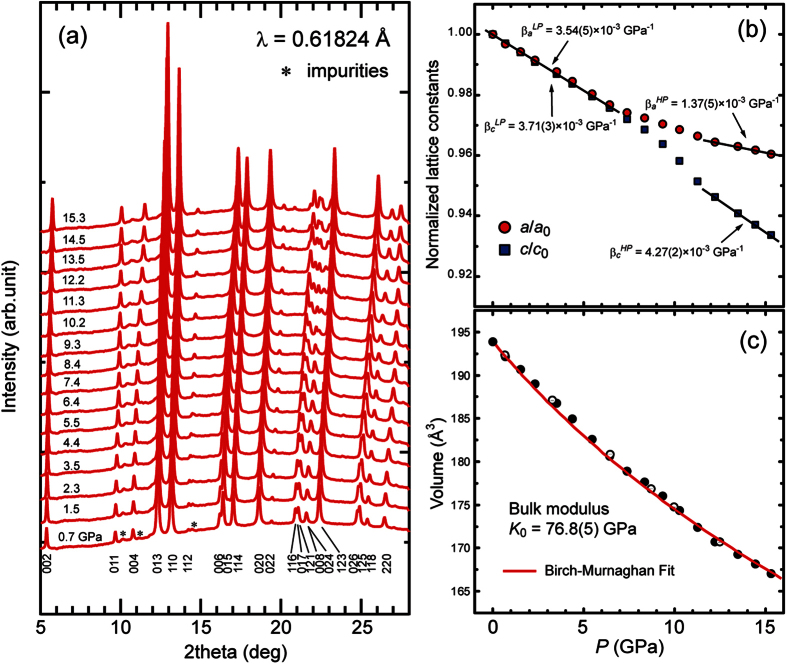
(**a**) Pressure evolution of synchrotron powder X-ray diffraction patterns collected from Sr_2_CoO_3_F at room temperature. The pressure is varied from 0.7 to 15.5 GPa. (**b,c**) Pressure dependence of *a*/*a*_0_ and *c*/*c*_0_ ratios, and volume. *a*_0_ and *c*_0_ are the lattice constants at ambient pressure. Open circles are the data during pressure release. Solid lines in the upper panel are guide for the eyes. Birch-Murnaghan fitting curves obtained by the equation (1) are represented with red solid line in the *V* vs *P* plots.

**Figure 3:**
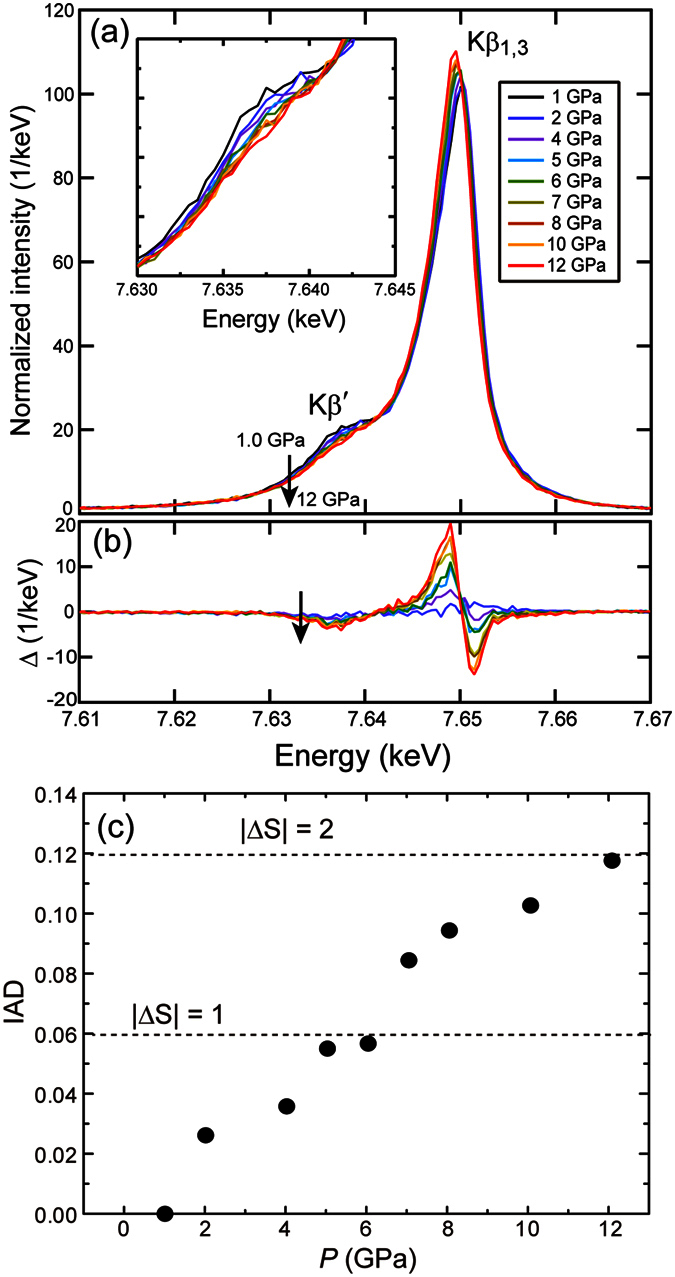
(**a**) Pressure evolution of Co *Kβ* emission spectra of Sr_2_CoO_3_F at room temperature. The inset expands the *Kβ′* satellite region. All the spectra are normalized to the spectral area from 7.61 to 7.67 keV. (**b**) The difference spectra (Δ) obtained by subtracting the 1 GPa spectrum from that at each pressure. (**c**) IAD values of the Co *Kβ* emission lines of Sr_2_CoO_3_F as a function of pressure.

**Figure 4:**
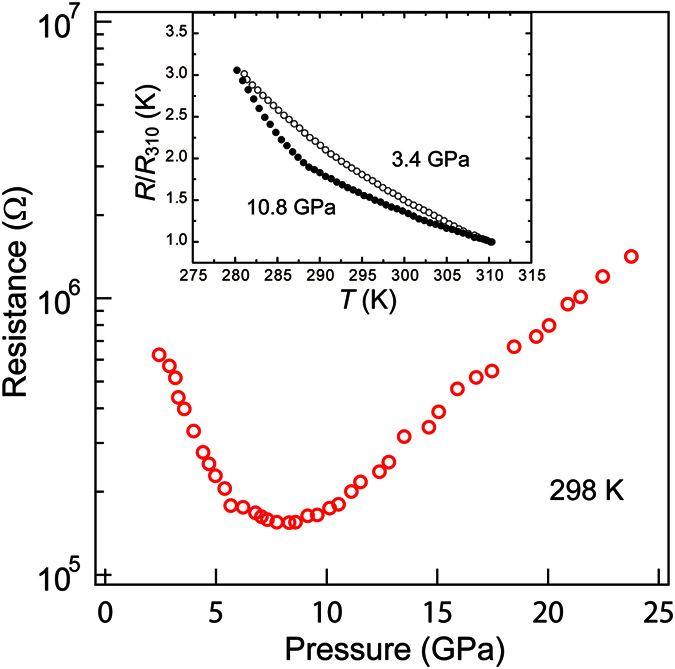
The pressure dependence of the electrical resistance (*R*) of Sr_2_CoO_3_F measured at 298 K. The inset displays the temperature dependence of the normalized resistance at 3.4 and 10.8 GPa.

The observed gradual spin state change is similar to the spin crossover behaviors of LaCoO_3_ under pressure, rather than the 1st order but incomplete spin transition to a mixed HS/LS state in 5-coordinated BiCoO_3_ with PbTiO_3_-type structure accompanied by the structural phase transition to GdFeO_3_-type structure. To investigate the correlation between the local structure and spin state change in Sr_2_CoO_3_F, Rietveld analysis was carried out for the SXRD data in a similar way to that described in our previous report^24^,^25^. To be specific, the atomic coordinates (*I*4/*mmm*) at ambient pressure reported in ref. 24 were used as the starting model. The atoms were placed at 4e (0 0 *z*) for Sr, Co, apical oxygen (O2), and F, and at 4c (0 1/2 0) for planar oxygen (O1). All the atomic coordinates and isotropic displacement parameters (*B*_iso_) were varied during refinements except for those of O2/F sites which were constrained into the same values. The site occupancies (*g*) for all the atoms were fixed to unity, but 0.5 for Co and O2/F because of site splitting and random distribution, respectively. The refinement for the data at each pressure converged with reasonable *R* indexes (*R*_wp_ < 1.0 and *R*_I_ < 2.0) and goodness-of-fit values(*S* < 0.5). Representative results of the Rietveld refinements against the 0.7 and 14.5 GPa data are presented in Supplementary Figure S2. The final refined crystallographic parameters in the *P* range of 0.7–14.5 GPa, and selected bond lengths and bond-valence-sum values at 0.7 and 14.5 GPa are summarized in Supplementary Tables S1 and S2, respectively. Local coordination environment around the Co ion at 0.7 and 14.5 GPa is illustrated in Fig. 1b.

**Figure 5:**
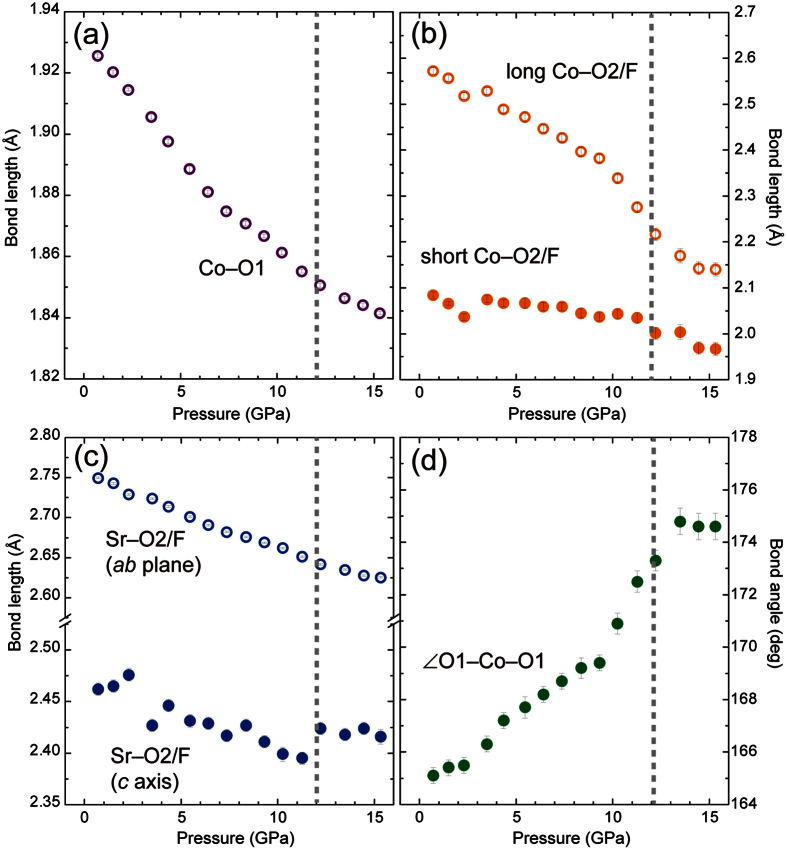
Pressure dependence of Co–O1, long and short Co–(O2/F), Sr–(O2/F) bond lengths, and O1–Co–O1 bond angle of Sr_2_CoO_3_F. Dash lines represent the pressure (*P*_s_ = 12 GPa) at which the HS state is completely depopulated.

Figure 5a,c show the pressure dependence of the Co–O1 and Sr–(O2/F) bond lengths in the basal plane. The pressure dependences of the two in-plane bonds correspond well to that of the *a* axis length: these linear compressibilities in 0 ≤ *P* ≤ 6.4 GPa are *β*_Co–O1_^*LP*^ = 4.0(1) × 10^−3^ GPa^−1^ and *β*_Sr–O2/F_^*LP*^ = 3.6(1) × 10^−3^ GPa^−1^, followed by significant reduction by nearly half in 12.2 ≤ *P *≤ 15.3 GPa. The value of *β*_Co–O1_^*MP*^ in the middle pressure region (7.4 ≤ *P* ≤ 11.3 GPa), where the anisotropic lattice contraction appears, is 2.7(1) × 10^−3^ GPa^−1^. The value of *β*_Co–O1_^*LP*^ is nearly close to the compressibility of the Co–O bond (4.8 × 10^−3^ GPa^−1^) for LaCoO_3_ in the pressure range where the HS state persists^12^,^13^ while the Co–O1 bond length at 14.5 GPa (1.8441(6) Å) is shorter than 1.891(1) Å for LaCoO_3_ with the full LS state at 5.99 GPa. It should be noted that the high value of *β*_Sr–O2/F_^*LP*^ in the *ab* plane results from the strong contraction of Co–O1 bond. Indeed, *β*_Sr–O2/F_^*LP*^ and *β*_Sr–O2/F_^*HP*^ along the *c* axis shown in Fig. 5c were 2.8(3) × 10^−3^ and 0.7(7) × 10^−3^ GPa^−1^, both much smaller than those in the basal plane. In Fig. 5b,d, the pressure dependences of the short and long Co–(O2/F) bond lengths along the *c* axis and the O1–Co–O1 bond angle in the basal plane are presented. At 0.7 GPa, the short and long Co–(O2/F) bond lengths are 2.073(8) and 2.573(8) Å, respectively. The bond length ratio of the latter to the former is 1.24. The O1–Co–O1 bond angle is 165.1(3)°. The two apical bonds revealed remarkable difference in linear compressibility even in the pressure range where the isotropic volume contraction occurs. The long Co–(O2/F) bond is extraordinarily compressible in 0 ≤ *P *≤ 9.3 GPa (*β*_2Co–O2/F_^*LP*^ = 8.5(4) × 10^−3^ PGa^−1^) and the compressibility is more than three times higher than that of the short Co–(O2/F) (*β*_1Co–O2/F_^*LP*^ = 2.5(2) × 10^−3^ PGa^−1^). Moreover, the long Co–(O2/F) bond length steeply decreased above 10 GPa, and then reached 2.143(14) Å at 14.5 GPa. The ratio of two apical bond lengths at 14.5 GPa was 1.09, much smaller than that at 0.7 GPa. Note that the high compressibility of the long Co–(O2/F) bond retained over the whole pressure range results in the anisotropic volume contraction above 6 GPa. Given the strong covalency in the Co–O bond leading to a BO_5_ square pyramid at ambient pressure, the short and long Co–(O2/F) bonds reflect the Co–O and Co–F bonding characters, respectively, as seen in related oxyhalides^32^,^33^,^34^,^35^. Therefore, the difference between the compressibilities of two apical bonds is derived from different bonding nature of fluoride and oxide ions. We found that the unusual shrinkage of the bond lengths was correlated with a gradual flattening of the CoO_4_ basal plane. The bond angle increased with pressure and reached a plateau at 174.5° above 13 GPa where the HS state was completely depopulated. It is obvious that the variation of the basal bond angle enhances/diminishes the effect of ionic-size reduction of Co ions on the compressibilities of the long/short apical bonds.

**Figure 6:**
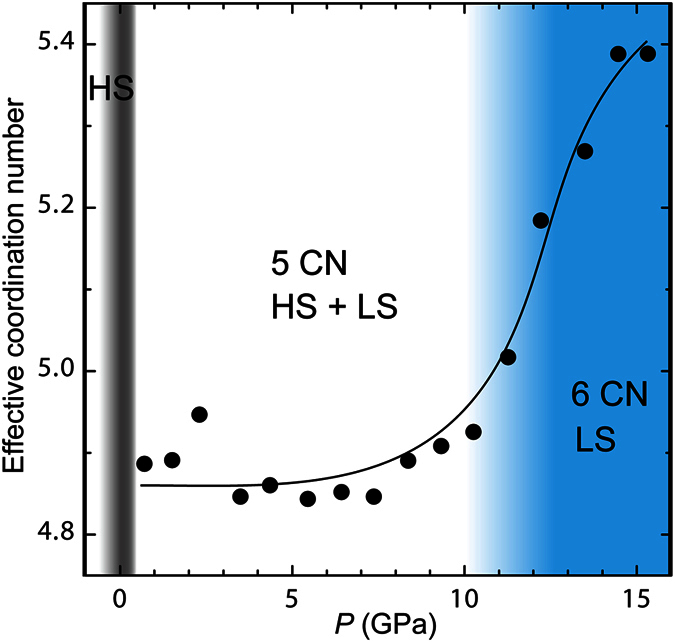
Pressure evolution of effective coordination number of CoO_5_F polyhedron. The coordination number increases from five to six above 10 GPa. The spin state change from the high spin (HS) to a low spin (LS) state occurs on the CoO_5_ pyramid in a wide pressure range, but a polyhedral transformation to CoO_5_F at around 11 GPa leads to the full conversion to the LS state at *P*_s_ = 12 GPa.

Sr_2_CoO_3_F exhibits the pressure-induced spin state change from the HS to LS state involving the transformation from square pyramid toward octahedron. However, the roles played by anions, especially fluoride, in spin state changes should be examined carefully. In the Co-centered coordination environment, the short Co–(O2/F) bond length at 14.5 GPa agrees well with the sum of 6-coordinate radii of LS Co^3+^ and O^2−^ ions (LS Co^3+^ = 0.685, O^2−^ = 1.26)^36^, consistent with the Co–O bonding character. But the other apical bond with Co–F bonding character is longer by 0.27 Å than the simple ionic model (F^−^ = 1.19)^36^. This indicates that the covalency in Co–F bond is not sufficient in comparison with Co–O bonds even above *P*_s_. To evaluate the influence of Co–F bond on the spin state change, we examined the effective coordination number (ECoN)^37^,^38^ of the Co metal center using VESTA^39^. For comparison, the ECoN’s for several related oxide materials with different coordination polyhedra are listed in Supplementary Table S3. We clearly see that the ECoN reflects the degree of polyhedral distortion. For example, both BiCoO_3_ and LaMnO_3_ with the GdFeO_3_ structure show smaller ECoN’s (5.77 and 5.23, respectively) than the ideal value. Furthermore, the Jahn-Teller effect on Mn^3+^ contributes much more to reduction in ECoN. As shown in Fig. 6, the ECoN value at 0.7 GPa is a reasonable value of 4.89 and remains almost unchanged up to 10 GPa, which suggests that the spin state change mainly results from the enhancement of the crystal-field splitting energy caused by the shrinkage of five covalent Co–O bonds. The depopulation of spins in *d*_*x*_^2^_−*y*_^2^ and *d*_*z*_^2^ orbitals also accounts for the gradual flattening of the CoO_2_ basal plane. Interestingly, the value of ECoN exhibits a rapid increase above 10 GPa, corresponding to the observed abrupt changes in the long Co–(O2/F) bond length and O1–Co–O1 bond angle. The ECoN value at 14.5 GPa is 5.39, higher than that for LaMnO_3_. In this context, the Co 3d and F 2p orbitals become hybridized just before the conversion to the LS state is completed, and thus the CoO_5_F polyhedron in the full LS state can be regarded as an octahedron rather than a square pyramid.

The difference between the spin crossover processes of Sr_2_CoO_3_F and BiCoO_3_ with pyramidal coordination can be rationalized by the mechanisms stabilizing the coordination geometry and the off-centering of Co sites. The XAS study by Sudayama *et al*. revealed that the strong Bi–O covalency results in smaller crystal field splitting than that in Sr_2_CoO_3_Cl isostructural with Sr_2_CoO_3_F^40^,^41^,^42^, which could be expected from the larger distance between the Co cation and the CoO_4_ basal plane for BiCoO_3_ (*D* = 0.74 Å) than those for Sr_2_CoO_3_Cl (*D* = 0.33 Å) and Sr_2_CoO_3_F (*D* = 0.25 Å). In Sr_2_CoO_3_F, it is likely that the primarily weak interaction between the Co and F ions and the relatively small distortion of CoO_5_ pyramid facilitate the gradual and complete spin state change from the HS state to the LS state. It is very rare that the coordination number changes in such a rigid solid crystalline phase, unlike organometallic molecules or coordination complexes with flexible metal-ligand bonds. Our observation of the gradual change in the coordination without a transition of the structural symmetry provides opportunities for new pressure-driven ligand-induced electronic state changes in other mixed anion compounds with similar coordination environments.

## Methods

A polycrystalline of Sr_2_CoO_3_F was prepared by high-pressure, high-temperature method using a belt-type high-pressure apparatus according to ref. 25. A stoichiometric mixture of in-house synthesized SrO_2_, SrF_2_ (99%, Rare Metallic CO., Ltd.), and Co (99.5%, Wako Pure Chemical Ind. Ltd.) was sealed in a Pt capsule, and heated under 6 GPa and 1900 °C for 30 min. Then, the sample was quenched to room temperature by turning off the heater before the pressure was released. The product was crushed into fine powders for high-pressure measurements described below.

High-pressure synchrotron powder X-ray diffraction (SXRD) study was conducted up to 15.3 GPa at room temperature at the beamline BL-18C of Photon Factory in High Energy Acceleration Research Organization (KEK), Japan. The Sr_2_CoO_3_F powders were loaded into a 160 um hole of a pre-indented rhenium gasket of a diamond anvil cell (DAC) with a pressure-transmitting medium of helium. Pressures were determined by the ruby-fluorescence method. The sample was irradiated using monochromatized X-ray beams (wave length, λ = 0.61825 Å). The powder diffraction data were recorded using an imaging plate (200 mm × 250 mm). Rietveld structure refinements were performed against the SXRD data using the RIETAN-FP program^43^.

XES measurements under high-pressures were carried out at a beamline BL39XU in SPring-8, Japan^44^. The Sr_2_CoO_3_F powders were loaded into a 160 um hole of a pre-indented beryllium gasket of a symmetric–type diamond anvil cell (DAC) with a pressure-transmitting medium of Daphne oil 7474. Applied pressures were varied in the range from 1.0 to 12 GPa. Pressures were determined by the ruby-fluorescence method. The incident beam was monochromatized by a Si 220 double-crystal monochromator and was focused into a spot of 9.5 (horizontal) × 1.8 (vertical) μm^2^ at the sample position with a Kirkpatrick-Baez mirror. The emitted X-rays were analyzed using a Si 620 spherically bent analyzer of 0.82 m radius. The energy spectra were measured by rotating the analyzer in the Bragg mode, synchronized with the detector motion so that the Rowland condition was maintained. The experimental resolution was 1.2 eV.

The temperature and pressure dependences of the electrical resistance were investigated using the four-probe method at various pressures up to 24 GPa. A Bassett-type DAC was used to generate pressures. Sample powers, a pressure-transmitting medium (NaCl), and platinum electrodes were loaded into a rhenium gasket covered with fine alumina powders for good electrical insulation. Pressures were determined by the ruby-fluorescence method.

## Additional Information

**How to cite this article**: Tsujimoto, Y. *et al*. Pressure-Driven Spin Crossover Involving Polyhedral Transformation in Layered Perovskite Cobalt Oxyfluoride. *Sci. Rep*. **6**, 36253; doi: 10.1038/srep36253 (2016).

**Publisher’s note:** Springer Nature remains neutral with regard to jurisdictional claims in published maps and institutional affiliations.

## Supplementary Material

Supplementary Information
