# Supplementary material for: Pressure-Driven Spin Crossover Involving Polyhedral Transformation in Layered Perovskite Cobalt Oxyfluoride
Source: Sci Rep. 2016 Nov 2;6:36253. doi: 10.1038/srep36253 (PMC5090247; doi:10.1038/srep36253)
Supplement: Supplementary Information [file srep36253-s1.pdf]

Supplementary Information

Pressure-Driven Spin Crossover Involving Polyhedral Transformation in Layered Perovskite Cobalt Oxyfluoride

Yoshihiro Tsujimoto,* Satoshi Nakano, Naoki Ishimatsu, Masaichiro Mizumaki, Naomi Kawamura, Takateru Kawakami, Yoshitaka Matsushita, and Kazunari Yamaura

Figure S1. (a) Room-temperature Co $K\beta$ emission spectra of Sr_2CoO_3F at 1 GPa under compression and decompression and (b) the difference spectrum. The intensity of a lower-energy satellite $K\beta'$ and the position of the main $K\beta$ line are returned in the decompression process. This indicates that the HS-to-LS state change is reversible.

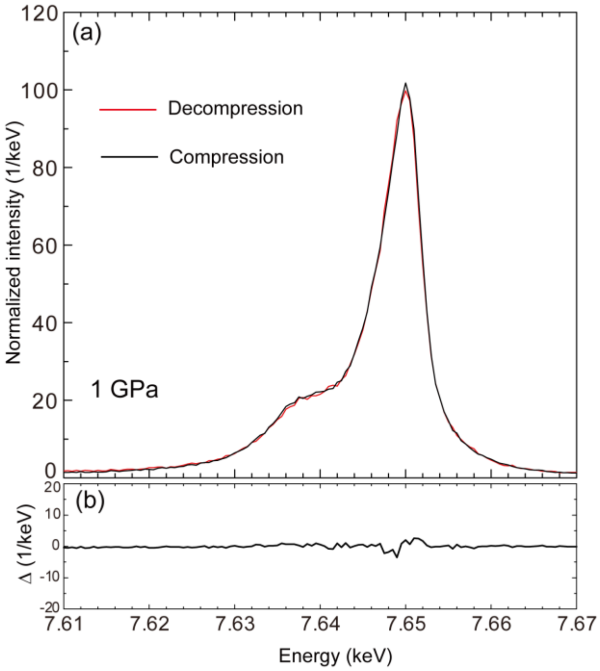

Figure S2. Experimental (crosses), calculated (upper line), and difference (bottom line) profiles at 0.7 and 14.5 GPa. Vertical lines represent the positions of the calculated Bragg reflections.

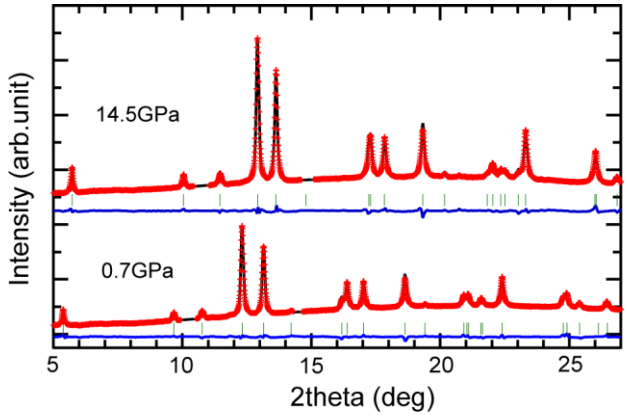

Table S1. Crystallographic Parameters Refined from Synchrotron X-Ray Diffraction Data Collected from Sr_2CoO_3F (Space Group, *I*4/*mmm*) at Various Pressures ($0.7 \leq P \leq 15.3$ GPa).

P	0.7	1.5	2.3	3.5	4.4	5.5
a /Å	3.81861(5)	3.80933(5)	3.79859(5)	3.78406(5)	3.77173(5)	3.75532(5)
c /Å	13.1768(3)	13.1387(3)	13.0962(3)	13.0425(3)	13.0013(3)	12.9474(3)
V /Å^3	192.141(5)	190.656(5)	188.970(5)	186.758(5)	184.955(6)	182.590(6)
Sr *z*	0.36317(6)	0.36348(7)	0.36298(6)	0.36258(7)	0.36339(7)	0.36301(7)
Sr B_{iso}/Å^2	0.32(3)	0.18(3)	0.27(3)	0.30(3)	0.19(4)	0.12(4)
Co *z*	0.9810(3)	0.9813(3)	0.9816(3)	0.9825(3)	0.9837(4)	0.9843(6)
Co B_{iso}/Å^2	0.94(8)	0.14(8)	0.19(8)	0.74(8)	0.66(9)	0.5(1)
O1 B_{iso}/Å^2	1.5(1)	1.3(1)	2.0(1)	0.5(1)	1.5(1)	0.31(1)
O2/F *z*	0.1763(3)	0.1758(3)	0.1739(3)	0.1764(3)	0.1752(4)	0.1752(4)
O2/F B_{iso}/Å^2	3.8(1)	5.2(1)	4.3(1)	4.4(1)	4.1(1)	6.2(1)
R_{WP} /%	0.5	0.56	0.57	0.61	0.66	0.64
R_I /%	1.3	1.7	1.52	1.14	1.64	1.37
S	0.29	0.34	0.35	0.38	0.42	0.41

P	6.4	7.4	8.4	9.3	10.2	11.3
a /Å	3.74217(5)	3.73167(5)	3.72510(5)	3.71732(4)	3.71069(5)	3.70247(5)
c /Å	12.8972(3)	12.8456(3)	12.8002(3)	12.7377(3)	12.6641(3)	12.5750(3)
V /Å^3	180.610(6)	178.880(6)	177.621(6)	176.017(5)	174.375(5)	172.382(5)
Sr *z*	0.36307(7)	0.36276(7)	0.36318(8)	0.36278(7)	0.36241(8)	0.36184(7)
Sr B_{iso}/Å^2	0.13(4)	0.01(1)	0.01(1)	0.35(4)	0.10(5)	0.21(4)

Co *z*	0.9849(4)	0.9856(5)	0.9862(5)	0.9864(5)	0.9883(6)	0.9904(7)
Co B_{iso}/Å^2	0.74(9)	0.93(9)	0.52(9)	1.24(8)	1.02(9)	1.04(8)
O1 B_{iso}/Å^2	1.6(1)	0.46(1)	2.2(2)	1.0(1)	1.4(1)	0.7(1)
O2/F *z*	0.1747(4)	0.1746(4)	0.1735(4)	0.1734(3)	0.1730(4)	0.1713(4)
O2/F B_{iso}/Å^2	4.2(1)	4.2(1)	4.1(1)	3.4(1)	3.7(1)	3.6(1)
R_{WP} /%	0.65	0.68	0.72	0.64	0.67	0.67
R_I /%	1.3	1.08	1.08	1.29	1.31	1.85
S	0.41	0.42	0.45	0.4	0.43	0.42

P	12.2	13.5	14.5	15.3
a /Å	3.69486(5)	3.68896(5)	3.68422(4)	3.67904(5)
c /Å	12.5057(3)	12.4364(3)	12.3864(3)	12.3396(3)
V /Å^3	170.727(5)	169.240(5)	168.127(4)	167.021(5)
Sr *z*	0.36251(8)	0.36221(8)	0.36173(8)	0.36219(9)
Sr B_{iso}/Å^2	0.09(4)	0.15(4)	0.19(4)	0.08(4)
Co *z*	0.9913(2)	0.993(1)	0.993(1)	0.993(1)
Co B_{iso}/Å^2	0.94(9)	1.08(9)	1.2(1)	0.82(8)
O1 B_{iso}/Å^2	2.1(1)	0.8(1)	1.2(1)	2.8(2)
O2/F *z*	0.1686(4)	0.1678(4)	0.1659(4)	0.1664(4)
O2/F B_{iso}/Å^2	3.4(1)	4.0(1)	4.3(1)	4.8(1)
R_{WP} /%	0.72	0.69	0.67	0.71
R_I /%	1.25	1.2	1.23	1.03
S	0.44	0.46	0.42	0.45

Space group: *I*4/*mmm*

B_{iso} represents the isotropic atomic displacement parameter. The values of B_{iso} and site occupancy (*g*) were independently constrained to the same value, respectively. The *g* values for Sr and O1 are 1, while those for Co, O2, and F are 0.5.

Table S2. Selected Bond Lengths and Bond-Valence-Sum Values for the 0.7 and 14.5 GPa Data.

cation	anion	Bond (Å)	BVS
0.7 GPa			
Sr	O1	2.6261(6) ×4	2.00
	O2/F1	2.462(6)	
	O2/F1	2.749(1) ×4	
Co	O2	1.9256(7) ×4	2.59
	O2/F1	2.073(8)	
	O2/F1	2.573(8) ×4	
14.5 GPa			
Sr	O1	2.5153(7) ×4	2.64
	O2/F1	2.424(6)	
	O2/F1	2.6277(7) ×4	
Co	O1	1.8441(6)×4	3.03
	O2/F1	1.969(14)	
	O2/F1	2.143(14)	

The BVS value of Sr at 0.7 GPa is 2.00, which agrees well with the value expected from the ionic model, while the BVS of the Co site is 2.59, much smaller than expected. This suggests that the Co center is under tension because of a non-covalent bond between Co and F ions. Similar trend is observed in structurally related Sr_2CoO_3Cl (BVS = 2.53) and $PbTiO_3$-type $BiCoO_3$ (BVS = 2.66). However, the BVS calculation for cobalt with full LS state at 14.5 GPa gives a consistent value of 3.03, which indicates that the local strain around the Co-centered polyhedron is released by the contraction of the long Co–(O2/F) with the Co–F bonding character. The Sr-centered polyhedron is composed of rigid ionic bonds with anions, and thus applying pressure raises the BVS value to 2.64.

Table S3. Comparison of Effective Coordination Number (ECoN) of Some Transition Metal Compounds with Different Coordination Polyhedra.

Compounds	Polyhedron	Space Group	Bond length	ECoN	Ref.
$LaCoO_3$	Octahedron	*R*-3*c*	Co-O×6 = 1.9318	6	4
$GdFeO_3$	Octahedron	*Pnma*	Fe-O×2 = 2.03648	5.90	5

$LaMnO_3$	Elongated octahedron	*Pbnm*	Fe-O×2 = 1.94744<br>Fe-O×2 = 1.94286<br>Mn-O×2 = 2.1800<br>Mn-O×2 = 1.9683<br>Mn-O×2 = 1.9055	5.23	6
$BiCoO_3$ (0.1 GPa)	Square pyramid	*P*4*mm*	Co-O×4 = 2.007<br>Co-O×1 = 1.747<br>Co-O×1 = 2.978	3.94	7
$BiCoO_3$ (3 GPa)	Octahedron	*Pnma*	Co-O×2 = 2.07427<br>Co-O×2 = 1.93346<br>Co-O×2 = 1.89952	5.77	7
$Sr_3Fe_2O_6$	Square pyramid	*I*4/*mmm*	Fe-O×5 = 1.9798<br>Fe-O×1 = 1.886	4.92	8
$Sr_2CuO_2Cl_2$	Square plane	*I*4/*mmm*	Cu-O×4 = 1.98580<br>Cu-Cl×2 = 2.8585	4.00	9
Sr_2CoO_3Cl	Square pyramid	*P*4/*nmm*	Co-O×4 = 1.9782<br>Co-O×1 = 1.894<br>Co-Cl×1 = 3.116	4.94	10
Sr_2NiO_3F	Square pyramid	*I*4/*mmm*	Ni-O×4 = 1.90953<br>Ni-(O/F)×1 = 2.02015<br>Ni-(O/F)×1 = 2.48012	4.98	11
Sr_2MnO_3F	Elongated octahedron	*I*4/*mmm*	Mn-O×4 = 1.89505<br>Mn-(O/F)l×2 = 2.281	4.62	12

References

1. a) Y. Tsujimoto, J. J. Li, K. Yamaura, Y. Matsushita, Y. Katsuya, M. Tanaka, Y. Shirako, M. Akaogi, E. Takayama-Muromachi, *Chem. Commun.* **2011**, *47*, 3263-3265; b) Y. Tsujimoto, C. I. Sathish, K-P. Hong, K. Oka, M. Azuma, Y. Guo, Y. Matsushita, K. Yamaura, E. Takayama-Muromachi, *Inorg. Chem.* **2012**, *51*, 4802-4809.
2. F. Izumi, K. Momma, *Solid State Phenom.* **2007,** *130*, 15-20.
3. N. Kawamura, N. Ishimatsu, H. Maruyama, *J. Synchrotron Rad.* **2009**, *16*, 730-736.

4. G. Thornton, B. C. Tofield, A. W. Hewat, *J. Solid State Chem.* **1986**, *61*, 301-307.
5. S. Geller, E. A. Wood, *Acta Cryst.* **1956**, *9*, 563-568.
6. J. Rodríguez, M. Hennion, F. Moussa, A. H. Moudden, L. Pinsard, A. Revcolevschi, *Phys. Rev B* **1998**, **57**, R3189-R3196.
7. K. Oka, M. Azuma, W-T. Chen, H. Yusa, A. A. Belik, E. Takayama-Muromachi, M. Mizumaki, N. Ishimatsu, N. Hiraoka, M. Tsujimoto, M. G. Tucker, J. P. Attfield, Y. Shimakawa, *J. Am. Chem. Soc.* **2010**, *132*, 9438-9443.
8. S. E. Dann, M. T. Weller, D. B. Currie, *J. Solid State Chem.* **1992**, *97*, 179-185.
9. L. L. Miller, X. L. Wang, S. X. Wang, C. Stassis, D. C. Johnson, J. Faber, C.-K. Loong, *Phys. Rev. B* **1989**, *41*, 1921-1925.
10. S. M. Loureiro, C. Felser, Q. Huang, R. J. Cava, *Chem. Mater.* **2000**, *12*, 3181-3185.
11. Y. Tsujimoto, K. Yamaura, T. Uchikoshi, *Inorg. Chem.* **2013**, **52**, 10211-10216.
12. Y. Su, Y. Tsujimoto, Y. Matsushita, Y. Yuan, J. He, K. Yamaura, *Inorg. Chem.* **2016**, *55*, 2627-2633.
